# Randomised clinical trial: pre-dosing with taribavirin before starting pegylated interferon vs. standard combination regimen in treatment-naïve patients with chronic hepatitis C genotype 1

**DOI:** 10.1111/j.1365-2036.2012.05188.x

**Published:** 2012-06-19

**Authors:** M Palmer, R Rubin, V Rustgi, Taribavirin Viral Kinetic Study Group

**Affiliations:** * Liver Center of Long IslandPlainview, NY, USA; † Digestive Healthcare of AtlantaAtlanta, GA, USA; ‡ Metropolitan Research, Georgetown Medical CenterFairfax, VA, USA

## Abstract

**Background:**

Combination therapy with the ribavirin (RBV) prodrug taribavirin (TBV) and pegylated interferon (PIFN) has produced lower rates of anaemia than with RBV and PIFN. Studies have demonstrated that the sharpest decline in viral load during TBV therapy occurs at Weeks 4 through 6, when TBV reaches steady-state blood levels.

**Aim:**

The current proof-of-concept study was conducted to examine whether first-order viral kinetics could be influenced by pre-dosing TBV to steady state before introducing PIFN.

**Methods:**

Therapy-naïve patients with chronic hepatitis C virus (HCV) genotype 1 (G1) were randomised to receive (i) TBV 600 mg BID monotherapy for 4 weeks followed by combination therapy with PIFN [pre-dosing arm (*n* = 23)] or (ii) TBV administered concurrently with PIFN [standard dosing arm (*n* = 19)].

**Results:**

More patients achieved undetectable virus or a ≥2-log_10_ reduction of HCV RNA at Week 4 in the pre-dosing vs. the standard dosing arm [33% vs. 22% (*P* = 0.497)]. There was also a trend towards greater reduction in mean log_10_ change in HCV RNA in the pre-dosing vs. the standard dosing arm, which was statistically significant at Day 1 [−0.34 ± 0.46 vs. 0.09 ± 0.32 (*P* < 0.003)] but not at other time points up to Week 24. No significant difference was observed in the rates of anaemia (haemoglobin <10 g/dL) between study arms (4.5% vs. 5.3%).

**Conclusions:**

Pre-dosing TBV prior to starting PIFN produces a trend towards improved efficacy although statistical significance was not reached in this small patient population. These results warrant larger clinical trials of TBV pre-dosing.

## Introduction

The addition of ribavirin (RBV) to interferon (IFN) accounts for an incremental advance in the treatment of chronic hepatitis C (HCV). While its mechanism of action is unclear, it is apparent that RBV improves sustained virological response (SVR) rates primarily by decreasing the likelihood of virological relapse after treatment discontinuation.^1^, ^2^ When weight-based RBV is added to pegylated IFN (PIFN), SVR rates improve by 25–30%.^3^, ^4^, ^5^ However, therapy is often compromised by dose-limiting anaemia which often prompts RBV dose reductions or discontinuations, which may lead to lower rates of SVR.^6^, ^7^ Furthermore, anaemia can significantly affect a patients' quality of life and treatment adherence.^8^ Ribavirin is an indispensible component of the current standard of care for patients with HCV genotypes 2 (G2) and 3 (G3)^9^ and is part of the backbone of triple therapy for patients with genotype 1 (G1) which includes the addition of a protease inhibitor – either boceprevir (BOC) or telaprevir (TVR) to PIFN.^10^ While triple therapy in G1 patients has been shown to increase SVR rates from approximately 45% to almost 80%, it is also associated with an increased incidence and severity of anaemia.^11^, ^12^, ^13^, ^14^

Taribavirin (TBV; 1-β-d-ribofuranosyl-1H-1, 2, 4-triazole-3-carboxamidine), previously known as viramidine, is a synthetic nucleoside (guanosine) analogue under investigation as a treatment for chronic hepatitis C.^15^ After oral administration, TBV is absorbed rapidly and acts as a liver-targeted prodrug of RBV; it is readily and extensively taken up by the liver and converted into its active metabolite, RBV. This allows for relatively high active drug levels within hepatocytes while simultaneously minimising systemic drug levels and thereby reducing the exposure of red blood cells (RBCs) to the potentially toxic effects of the drug.^15^, ^16^, ^17^, ^18^, ^19^, ^20^

Significantly lower rates of anaemia were observed among patients who were treated with PIFN plus TBV compared with those treated with PIFN plus weight-based doses of RBV in the VISER 1 [Taribavirin (Viramidine) Safety and Efficacy vs. Ribavirin] study.^21^

Viral decay kinetic data from an interim analysis of this study conducted in patients with HCV treated with TBV plus PIFN, showed the sharpest decline in HCV RNA within the first 4 weeks of therapy.

The goal of the current analysis was to compare the Week 4 viral load in patients receiving 4 weeks of TBV monotherapy followed by PIFN plus TBV combination therapy vs. simultaneous initiation of PIFN plus TBV. Notably, this study was terminated early based on the VISER 1 study results, which failed to show non-inferiority of SVR rates with PIFN plus TBV vs. PIFN plus weight-based RBV.^21^

## Materials and Methods

### Patients

This study enrolled treatment-naïve G1 patients with compensated liver disease secondary to chronic HCV at 10 investigational sites throughout the United States; target enrolment was 100 patients for this proof-of-concept study. Compensated liver disease was defined as normal prothrombin time, serum albumin and bilirubin levels and no history or evidence of bleeding oesophageal varices, ascites or hepatic encephalopathy.

All patients were screened according to clinical history, physical examination, laboratory testing (biochemistry, haematology, urinalysis, serum pregnancy test, urine drug screen), 12–lead electrocardiogram, chest radiograph and liver biopsy evaluation.

Criteria for inclusion in this study were (i) Adults aged 18 through 70 years; (ii) body weight >61 kg and ≤87.3 kg; (iii) genotype 1; (iv) history of alanine aminotransferase elevation in the preceding 6 months or elevated alanine aminotransferase during the screening period; (v) platelet count ≥90 000/mm^3^, absolute neutrophil count ≥1200/mm^3^, and haemoglobin ≥12.0 g/dL for women and ≥13.0 g/dL for men; (vi) creatinine clearance >70 mL/min; (vii) glycosylated haemoglobin (A1c) levels ≤8.5% in diabetic patients; (viii) normal or adequately controlled thyroid-stimulating hormone levels on prescription medication; (ix) alpha-fetoprotein levels within normal limits or hepatocellular carcinoma ruled out within 6 months prior to study entry; (x) Patients with chronic HCV infection documented by a positive HCV antibody and a detectable HCV RNA (xi) liver biopsy within 3 years prior to screening showing results consistent with chronic HCV infection interpreted by a local pathologist; and (xii) inability to conceive or active use of appropriate birth control methods.

Patients were excluded from the study if they had a positive HIV or hepatitis B surface antigen serology; severe psychiatric or neuropsychiatric disorders (e.g. severe depression, history of suicidal ideation); significant ischaemic or unstable heart disease; significant metabolic, haematologic, pulmonary, gastrointestinal, neurological, renal, urologic, endocrine, ophthalmologic (including retinopathy) or immune-mediated disease; history of thalassemia or other haemoglobinopathies; organ or bone marrow transplantation; chronic use of immunosuppressive medications (>30 days), including steroids, in doses equivalent to ≥10 mg of prednisone 30 days before or anytime during the study; recent history of alcoholism or illicit drug use; malignancy diagnosed and/or treated within the preceding 5 years; and breastfeeding or a positive pregnancy test result at any time during the study.

Written informed consent was obtained from all patients prior to enrolment in this study. This trial was registered at Clinicaltrials.gov identifier number NCT00305383.

### Study design

**Figure 1:**
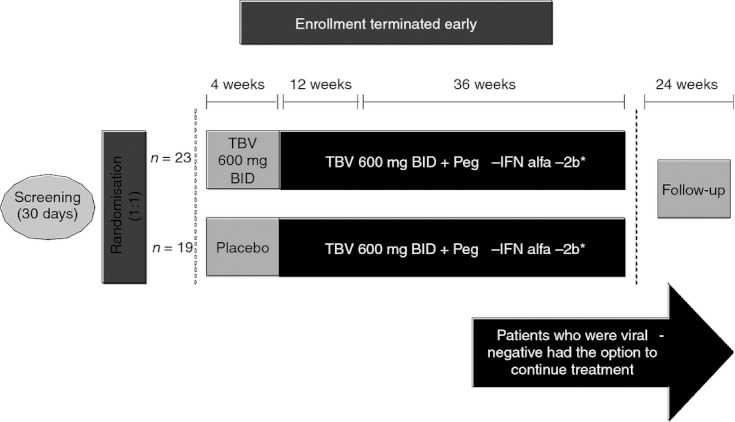
Study protocol. HCV, NGI SuperQuant; sensitivity to 39 IU/mL. *Peg-IFN alfa-2b 1.5 μg/kg/week.

This phase 2b, multicentre trial included a 4-week, double-blind, monotherapy period, followed by an open-label 48-week combination therapy period. Study patients were randomised in a 1:1 ratio to receive either (i) TBV (Valeant Pharmaceuticals International, Aliso Viejo, CA, USA) 600 mg orally twice daily for 4 weeks followed by combination therapy with TBV 600 mg orally twice daily plus PIFN alfa-2b (PegIntron Redipen, Schering Corp., Kenilworth, NJ, USA) 1.5 μg/kg per week subcutaneously for 48 weeks (predose arm, *n* = 23) or (ii) placebo orally twice daily for 4 weeks followed by combination therapy with TBV 600 mg orally twice daily and PIFN 1.5 μg/kg per week subcutaneously for 48 weeks (standard dosing arm, *n* = 19) (Figure 1).

The use of any haematopoietic growth factors (e.g. erythropoietin, granulocyte colony-stimulating factor, thrombopoietin) was prohibited during the course of the study. All protocols were approved by the local institutional ethics boards and were in accordance with the declaration of Helsinki for the investigation of human subjects.

### Data collection and statistical analysis

Demographic information, vital signs and baseline characteristics were summarised for all randomised patients who took at least one dose of the study drug. Descriptive statistics were used for continuous variables, and percentages were used for categorical measurements. No statistical tests were performed on demographic variables.

All patients underwent reassessment with clinical interview, physical examination and laboratory testing at various time points (combination therapy Days 1 and 2 and Weeks 1, 4, 8, 12 and 24). All laboratory tests were performed at a central facility (Covance Central Laboratory, Indianapolis, IN, USA). The primary efficacy end point was the proportion of patients who experienced, an undetectable plasma HCV RNA <39 IU/mL [NGI SuperQuant (National Genetics Institute, Los Angeles, CA, USA); sensitivity to 39 IU/mL] or a ≥2-log_10_ drop from baseline after 4 weeks of combination therapy. Secondary efficacy end points included the proportion of patients who experienced a virological response at combination therapy Week 24 and mean log_10_ change from baseline viral load at various therapy time points (combination therapy Days 1 and 2 and Weeks 1, 4, 8, 12 and 24).

The primary analysis population was the intent-to-treat population, (ITT) which included all randomised patients who received at least one dose of study drug. The per-protocol population (PPP) was also analysed.

The incidence of adverse events (AEs) was monitored during the study after the first dose of study drug and continuing throughout the study period. For the purposes of AE monitoring, anaemia was defined as a haemoglobin <10 g/dL, and the difference between treatment groups for the proportion of patients with haemoglobin <10 g/dL at any time during the treatment was tested using logistic regression model with treatment and baseline haemoglobin as independent variables. All randomised patients who received at least one dose of study drug were included in the analysis.

## Results

### Patients

A total of 42 patients met eligibility requirements and were randomised to pre-dosing (*n* = 23) or standard dosing arm (*n* = 19). All patients in the study were therapy-naïve, G1 and most had a high viral load (≥800 000 IU/mL). Data from the phase 3 VISER 1 trial of TBV plus PIFN became available during the conduct of this study.^10^ Although the target study population for this proof-of-concept study was 100 patients, the VISER 1 data failed to show non-inferiority of this combination compared with weight-based RBV plus PIFN and the current study was terminated prior to enrolment completion. Baseline patient demographics of the 42 patients are summarised in Table 1.

**Table 1:** Baseline patient demographics

	Pre-dose taribavirin (*n* = 23)	Standard dose taribavirin (*n* = 19)
Average age, year	50	48
Male gender (%)	57	63
White (%)	83	95
Mean weight (kg)	77	76
Mean body mass index (kg/m^2^)	26	26
HCVRNA mean (log_10_IU/mL) (s.d.)	6.78 (range 3.78–8.19) (0.47)	6.66 (range 4.11–7.94) (0.49)
Metavir% F1–F2	87	74
Metavir% F3–F4	13	26

### Efficacy

**Figure 2:**
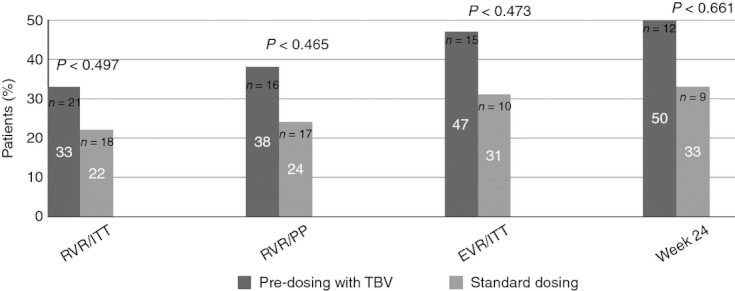
Undetectable virus or ≥2-log drop in viral load. RVR, rapid virologic response; ITT, intent-to-treat population; PP, per-protocol population; EVR, early virological response.

Four weeks after initiation of combination therapy, a greater proportion of patients achieved undetectable viral load (HCVRNA < 25 IU/dL) or a ≥ 2-log_10_ reduction of HCV RNA in the pre-dosing vs. the standard dosing arm in the ITT (33% *n* = 21 vs. 22% *n* = 18) and the PPP (42 patients) (38% vs. 24%). However, this difference did not reach a level of statistical significance (*P* < 497). A similar trend was observed for patients in the pre-dosing vs. standard dosing arm for achieving an early virological response at Week 12 (47% *n* = 15 vs. 31% *n* = 10) (*P* < 0.473) and an end-of-treatment response at Week 24 (50% *n* = 12 vs. 33% *n* = 9) (*P* < 0.661) (Figure 2).

**Figure 3:**
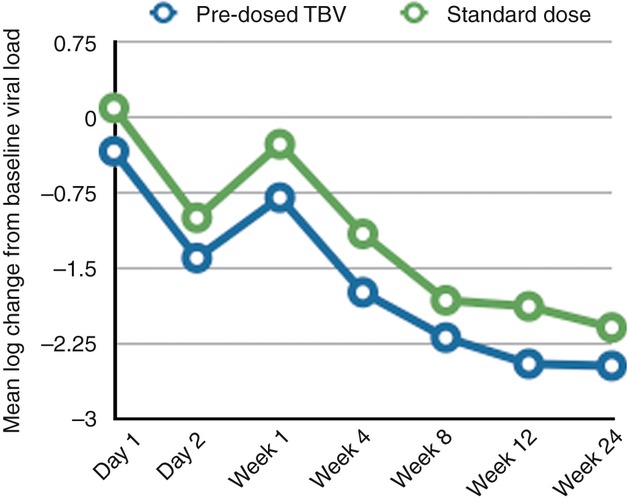
Mean log change from baseline viral load at key therapy time points pre-dosed vs. standard dosed TBV – viral kinetic curve.

Mean log_10_ reductions from baseline in HCV RNA were significantly greater in the pre-dosing vs. the standard dosing arm at Day 1 [−0.34 ± 0.46 vs. 0.09 ± 0.32 (*P* ≤ 0.003)] (Table 2, Figure 3). There was also a nonsignificant trend towards greater reductions in the predosing vs. the standard dosing arm at other time points up to Week 24.

**Table 2:** Mean log change from baseline viral load at key therapy time points pre-dosed vs. standard dosed TBV

	Pre-dosing TBV 600 mg BID			Standard dosing TBV 600 mg BID			
*n*	m	STD	*n*	m	STD	*P* value
CT Day 1	17	–0.34	0.46	17	0.09	0.32	≤0.003
CT Day 2	16	–1.40	0.69	16	–1.00	0.92	≤0.174
CT Week 1	17	–0.80	0.83	16	–0.27	0.79	≤0.070
CT Week 4	17	–1.74	1.28	17	–1.16	1.27	≤0.194
CT Week 8	15	–2.19	1.81	16	–1.82	2.03	≤0.597
CT Week 12	15	–2.45	2.12	16	–1.88	2.19	≤0.468
CT Week 24	25	–2.47	2.31	13	–2.09	2.21	≤0.662

This study was terminated early due to the results obtained from the Viser 1 clinical trial and collection of data past this time point was not performed. After study termination, treatment decisions were made on an individualised basis at the investigators discretion and data were not collected or analysed.

### Safety

The incidence of anaemia was similar between treatment arms (4.3% of the pre-dosing arm vs. 5.0% of the standard dosing arm), and there was no significant difference in the proportions of patients experiencing a haemoglobin decline >25% (7% of the pre-dosing arm vs. 13% of the standard dosing arm) (Table 3). Furthermore, there were no significant differences in the frequencies of other AEs between treatment arms, although there was a nonsignificant trend towards more diarrhoea and rash with longer TBV exposure (Table 4). There were no treatment terminations or dose reductions in either arm.

**Table 3:** Incidence of anaemia and haemoglobin decline in pre-dosed vs. standard dose TBV through Week 24 of therapy

Dosing TBV	Pre-dosing TBV (*n* = 23) %	Standard dosing TBV (*n* = 19) %
Anaemia (Hgb < 10 g/dL)	4.3	5.0
Haemoglobin decline	7.0	13.0

**Table 4:** Summary of all treatment-emergent adverse events

	Pre-dosing TBV (*n* = 23) %	Standard dosing (*n* = 19) %
Diarrhoea	30.4	15.8
Flatulence	13.0	10.5
Haemorrhoids	4.3	10.5
Nausea	21.7	26.3
Chills	13.0	21.1
Fatigue	43.5	42.1
Influenza-like illness	39.1	42.1
Dehydration	8.7	10.5
Headache	39.1	47.4
Anxiety	17.4	10.5
Depression	8.7	26.3
Dyspnoea	8.7	15.8
Rash	26.1	10.5
Hypertension	13.0	15.8

## Discussion

This study demonstrates that 4 weeks of TBV monotherapy prior to starting PIFN leads to a steeper decline in HCV RNA with no difference in anaemia rates. While this trend is relatively consistent over the 24-week experimental period, the difference does not reach a level of statistical significance. This observation, combined with the relatively high variance in viral load at key time points, suggests that a larger study population is required to further delineate the effects of TBV pre-dosing on phase 1 viral kinetics. Alternatively, the variance in the viral decline may be a function of both the impact of having TBV at steady state when PIFN is introduced into therapy and/or increased conversion of TBV to RBV due to longer exposure.

Studies have demonstrated that compared with patients treated with PIFN alone, patients pre-dosed with RBV display enhanced induction of the IFN-related genes (STAT1, ISGF3, IRF-7 and the interferon receptor) that are initially involved in the IFN-signalling cascade. In this manner, RBV may make hepatocytes more receptive to the action of IFN and may boost endogenous IFN production.^22^

Merli and colleagues evaluated the safety and efficacy of pre-dosing RBV 8 weeks prior to starting 48 weeks of combination PIFN/RBV in patients who developed recurrent HCV after liver transplantation.2011 They demonstrated that RBV priming both enhanced efficacy and improved adherence, and proposed that by administering medications in this sequential manner, patients have time to adjust to the side effects of each medication. In their study, 15% of patients necessitated RBV dose reductions compared with approximately 40% of patients (range of between 24% and 92%) when PIFN/RBV was administered concurrently in studies evaluating similar patient populations post transplant.^24^, ^25^, ^26^, ^27^, ^28^ Their study also demonstrated that RBV priming results in improved SVR rates compared with results of published trials performed using standard combination therapy −46% vs. 29% respectively.^23^, ^26^ While our study was discontinued prior to collection of SVR data, mean log_10_ reductions from baseline in HCV RNA were significantly greater in the pre-dosing vs. the standard dosing arm at Day 1 [−0.34 ± 0.46 vs. 0.09 ± 0.32 (*P* ≤ 0.003)]. Merli *et al*. also found that the extent of HCV RNA reduction pre-treatment was found to be a predictor of on-treatment response, a result consistent with the findings from our study.

Tox and colleagues pre-treated twenty patients with chronic HCV and normal ALT with 4 weeks of RBV followed by combination therapy of RBV and low-dose PIFN (50 mcg SQ q weekly) for 44 weeks, and found similar SVR rates when compared with historical controls treated with simultaneously initiated RBV and full dose PIFN.^29^ Larger clinical trials need to be conducted to confirm these intriguing results, especially in poorly IFN-tolerate populations.

Administered as monotherapy, RBV can normalise ALT levels,^30^, ^31^, ^32^, ^33^ improve liver histology,^30^, ^34^ and temporarily decrease HCVRNA levels by approximately 1.0 log_10_ IU/mL.^35^ RBV may act as a lethal mutagen increasing the incidence of viral mutations thus limiting HCV replication via derror catastrophe.^36^ While RBVs mutagenic potential on HCV remains debated,^37^ RBV monotherapy has been associated with an increase in the mutation rate in the NS5A and NS5B regions of HCV which correlated with SVR rates when patients were retreated with combination therapy.^38^ In fact, Ogawa and colleagues postulated that 4 weeks of RBV monotherapy prior to starting IFN/RBV combination therapy, stimulates the host immune system and promotes HCV gene mutations resulting in improved IFN efficacy.2009

Although the therapeutic mechanisms of RBV in patients with HCV remain unclear, the mechanisms by which RBV can induce dose-limiting haemolytic anaemia are better understood. After entering the circulation, a significant portion of RBV is transported into RBCs and metabolised into various phosphorylated derivatives.^17^ Because of the lack of phosphatase activity in RBCs, these phosphorylated metabolites of RBV are trapped intracellularly and accumulate over time, leading to depletion of intracellular adenosine triphosphate, impaired adenosine triphosphate–dependent oxidative respiration and impaired membrane integrity, culminating in haemolysis.^40^ Anaemia is a common side effect of RBV therapy. Most patients experience a rapid decline of 2–3 g/dL in haemoglobin during the first 4 weeks of initiating RBV therapy.^3^, ^4^, ^30^ and approximately 50% of patients on combination PIFN/RBV experience a haemoglobin decline of ≥4 g/dL.^41^

RBV-associated haemolytic anaemia is the primary reason for dose reductions and treatment termination in patients taking PIFN/RBV.^42^ The addition of either BOC or TVR to PIFN/RBV in the treatment of patients with G1 HCV increases the incidence of anaemia. In clinical trials of BOC triple therapy vs. a control group treated with PIFN/RBV, 49% of patients experienced anaemia, defined as a haemoglobin level <10 g/dL, and 26% of patients required RBV dose reduction due to anaemia vs. 29% and 13%, respectively, in controls. Erythropoietin alpha (EPO) was administered to patients (at the investigators discretion) in 43% of patients on BOC triple therapy vs. 24% of controls respectively. In patients on BOC triple therapy, 3% required blood transfusions and approximately 3% discontinued therapy early due to anaemia vs. <1% and <1% in controls respectively.^13^, ^14^

In clinical trials of TVR triple therapy vs. a control group treated with PIFN/RBV controls, 32% of patients experienced anaemia and 22% of patient's required RBV dose reduction due to anaemia vs. 15% and 9% in controls respectively. EPO use was prohibited in TVR-based studies. In patients on TVR triple therapy 4.6% required blood transfusions and 2% discontinued therapy early due to anaemia vs. 1.6% and 0.5% in controls respectively.^11^, ^12^

Studies have shown that G1 patients with inosine triphosphatase (ITPA) deficiency - a benign inherited enzymopathy in which inosine triphosphate accumulates in red blood cells, are protected from RBV-associated haemolytic anaemia. These patients are less likely to require RBV dose reductions.^43^, ^44^ Our study was conducted prior to the discovery of the significance of ITPA deficiency. It would be warranted to test patients enrolled in future trials of TBV or other RBV prodrugs for ITPA deficiency.

In contrast to RBV, administration of TBV, results in high hepatic RBV levels and limits RBV metabolite accumulation in RBCs. Indeed, pharmacokinetic studies in nonhuman primates revealed that TBV-derived RBV is concentrated in the liver in an amount that is 38% higher than that seen after administration of RBV.^16^ Furthermore, phosphorylated TBV metabolites were not detected in monkey RBCs after single and multiple TBV doses, suggesting that TBV cannot enter RBCs until it is converted to RBV.^19^ These preclinical results are supported by data from two phase 3 studies, - (Viramidines's Safety and Efficacy vs. Ribavirin) VISER 1 and VISER 2, which demonstrated that TBV was significantly less likely than RBV to cause anaemia (5%–6% vs. 22%–24%).^21^, ^45^

Although both studies showed that TBV was associated with lower rates of anaemia, at a fixed- dose of 600 mg twice daily, (as was administered in our study,) SVR rates were lower (38%–40%) with TBV/PIFN than with PIFN/RBV (52%–55%).^21^, ^45^ However, there was a trend towards greater efficacy among patients with higher exposure to TBV on the basis of body weight, and the sharpest decline in viral load occurred at Weeks 4 through 6,^21^ presumably when TBV activity reached steady state. In fact, patients from North America and Europe met non-inferiority for both the total as well as for the subset of patients weighing <75 kg with SVR rates of 51 vs. 56% and 62 vs. 60% respectively.^21^ Indeed, results of a randomised active-controlled, parallel-group study of 278 treatment-naïve G1 patients confirmed that patients treated with weight-based taribavirin and PIFN have equal efficacy yet less anaemia compared with those treated with weight-based ribavirin and PIFN.^46^ Specifically while there were no statistical differences in SVR rates, the incidence of anaemia was significantly lower (*P* < 0.05) in patients receiving both 20 and 25 mg/kg/day TBV treatment groups (13.4% and 15.7%, respectively) compared with RBV (32.9%). It was concluded from this trial that the 25 mg/kg/day dose of TBV resulted in the optimal combination of treatment efficacy and safety. Since our study was completed prior to the publication of these results, a larger trial pre-dosing TBV at 25mg/kg/day is warranted. In addition to the small sample size and lack of weight-based TBV, as TBV is converted to RBV by adenosine deaminase (ADA), polymorphisms of ADA could develop, further contributing to the lack of statistically significant results found in our study.

While our study was not designed to address the incidence of anaemia in TBV compared with RBV, the rate of anaemia in both the pre-dosed TBV arm and the standard start TBV arm had extremely low anaemia rates −4.3% and 5% respectively. Furthermore, no patient underwent TBV dose reductions or terminations, or required a blood transfusion. The use of EPO was prohibited in the current clinical trial. The EPO has been observed to improve quality of life and adherence to PIFN/RBV treatment while lessening the need for RBV dose reductions in patients developing anaemia on combination therapy.^8^ Still, other studies have yielded conflicting results as to the impact of EPO on SVR.^47^, ^48^ It should be underscored that EPO is not approved by the FDA for use in combination with PIFN/RBV. Furthermore, EPO has been associated with its own unique adverse events, and the addition of EPO to treatment regimens increases both the complexity and cost of treating patients with HCV.

For patients with G1 HCV receiving triple combination therapy with a protease inhibitor plus PIFN/RBV, the protease inhibitor cannot be dose reduced or used without RBV. Thus, if RBV requires permanent discontinuation, BOC or TVR would also need to be permanently discontinued due to the risk of developing viral rebound and/or drug resistance. Given the added concerns of anaemia developing during triple combination therapy, studies are needed to determine the safety and efficacy of TBV instead of RBV as a component of triple therapy.

## Conclusion

In conclusion, a 4-week course of TBV prior to initiating combination therapy with TBV plus PIFN for chronic HCV infection resulted in a trend towards rapid and early decline in HCV RNA and low incidence of anaemia. Based on these data, a larger study examining pre-dosing of TBV is warranted to assess the impact of TBV steady-state concentration on phase 1 viral decline, SVR and anaemia. Furthermore, since the addition of a protease inhibitor to PIFN/RBV for patients with G1 HCV has shown to increase the incidence and severity of anaemia, studies evaluating TBV in combination with PIFN and a protease inhibitor are also needed. Lastly, TBV should also be studied in patients at increased risk for the development of anaemia with RBV, such as those with cirrhosis, renal insufficiency, haemoglobinopathies and recurrent HCV after liver transplantation.
